# How to disagree better: utilizing advocacy-inquiry techniques to improve communication and spur behavior change

**DOI:** 10.1017/ash.2023.457

**Published:** 2023-11-06

**Authors:** Alyssa Y. Castillo, Jeannie D. Chan, John B. Lynch, Chloe Bryson-Cahn

**Affiliations:** 1 Division of Infectious Diseases, Department of Medicine, University of Colorado School of Medicine, Aurora, CO, USA; 2 Department of Pharmacy, Harborview Medical Center, University of Washington School of Pharmacy, Seattle, WA, USA; 3 Division of Allergy & Infectious Diseases, Department of Medicine, University of Washington School of Medicine; Seattle, WA, USA

## Abstract

The ability to provide feedback to a colleague is a key skill required for professional growth and patient safety. However, these conversations are limited by time constraints, differences in values, and a culture of “noninterference.” This *advocacy-inquiry-identify-teach* framework creates an organized approach to initiating successful “challenging” conversations with peers.

## Introduction

Challenging conversations are ubiquitous in medicine, perhaps none more so than providing unsolicited feedback to a colleague with whom there is clinical disagreement. These conversations are common in antimicrobial stewardship, as many programs utilize prospective audit-and-feedback—the process of reviewing real-time antimicrobial use and highlighting opportunities for improvement in the clinical care provided by a peer (eg, de-escalation of unnecessarily broad antibiotics or optimization of duration of therapy).^1^ For this reason, the context of antimicrobial stewardship will be used to demonstrate a framework to approach challenging conversations, though this structure can be applied to any domain or specialty.

Fostering a productive and collegial conversation is challenged by several factors, including perceived and real differences in training and expertise between the steward and provider, lack of alignment in values and priorities (eg, a steward’s emphasis on antibiotic appropriate use versus a clinician’s need for efficiency on a busy clinical service), and the emotional beliefs that often inform antibiotic prescribing habits.^2^ In addition, providers often abide by an unspoken culture of “noninterference”—the mutual posture of not questioning the decision-making autonomy of colleagues—that limits their willingness to intervene on the prescribing habits of peers.^3^

Despite these challenges, the benefits of creating a positive and collegial conversation are myriad. Most immediately, a successful stewardship intervention improves the quality and safety of care for patients. Long-term, productive conversations facilitate strong relationships between peers and create a safe space for teaching—thus reducing the need for repeated interventions and promoting future collaboration.

## Elements of a productive conversation & the advocacy-inquiry framework

Fostering a productive conversation requires attention to delivery and content, recognizing that a successful “difficult” conversation still produces a positive learning experience. First, the conversation must be non-confrontational to avoid triggering defensiveness. Second, the conversation must be direct and concise to maintain the colleague’s focus and attention—especially given that these conversations often occur in busy clinical settings. Third, the steward must accurately identify and teach to a colleague’s specific knowledge gap to avoid appearing condescending or patronizing by sharing concepts that are already known. Finally, a productive conversation creates a sense of shared ownership of a patient, with emphasis on the function of both individuals as members of one team with collective responsibility.

The *advocacy-inquiry* framework serves as one approach to cultivate a constructive conversation. This methodology originated in the business and organizational behavior literature and was later adopted in the medical simulation sphere as part of “debriefing with good judgment”—an approach designed to encourage open dialogue, reflective thinking, and accurate assessment of a learner’s thought process.^4–6^ In this framework, an *advocacy* statement is utilized to make an objective observation and is followed by an *inquiry* designed to reveal a learner’s assumptions and identify the “invisible frames” that inform their actions.^5^

Psychological safety—defined as “a shared belief that the team is safe for interpersonal risk taking” and a sense of “confidence that the team will not embarrass, reject, or punish someone for speaking up”—is of particular importance in highly functioning clinical teams.^7,8^ Notably, the objective and candid nature of *advocacy–inquiry* statements promotes a culture of psychological safety by encouraging strategies that are associated with safe learning environments—including normalizing discussion of mistakes, fostering curiosity, seeking new perspectives, and recognizing the valuable contributions of all team members.^8^

## Steps 1 and 2: Advocacy-inquiry

**Advocacy.** The *advocacy* statement highlights a discrepancy between a clinical situation and a perceived inconsistent clinical response with factual observations. Successful advocacy statements often begin with self-oriented language: “*I noticed…”* or “*I saw…*.”

**Inquiry.** The *inquiry* subsequently probes this discrepancy with an open-ended question to query the thought process yielding the colleague’s decision or action. Successful inquiries often begin with *“Can you help me understand…?” “Can you share more about…?”* or *“Can you teach me…?”*

A paired *advocacy-inquiry* statement is a powerful way to open a conversation. It is concise and direct, highlighting a specific issue in two sentences. Despite its brevity, it is also impartial—effectively calling attention to this discrepancy without making assumptions about what occurred, why it happened, or who is responsible for a decision. Finally, it rapidly opens the door for bidirectional conversation, promoting a sense of collegiality and shared decision-making regarding a patient’s care.

Additionally, an *advocacy-inquiry* statement allows stewards to open the conversation with a posture of humility. Antimicrobial stewards are often not at the bedside and therefore make recommendations based on chart review alone; as such, the open-ended *inquiry* creates space for stewards to glean new information from the provider that may influence their recommendations (eg, undocumented symptoms).

The juxtaposition of a clinical situation with a perceived incompatible clinical action through a paired *advocacy-inquiry* statement may be sufficient for a colleague to self-identify an opportunity for improvement. Thus, pausing after the *advocacy-inquiry* statement allows a colleague to spontaneously correct their error; in these cases, stewards may simply affirm the colleague’s revised action, and the subsequent *identify-teach* intervention may not be required.

## Steps 3 and 4: Identify-teach

**Identify.** The third step, *identify,* is active listening. The goal of this step is to not simply hear the colleague’s response, but to understand the thought process that yielded their clinical decision. Correctly diagnosing the underlying reason guiding their actions allows the steward to identify what concepts to teach to specifically address the colleague’s cognitive error(s).

**Teach.** In this final step, the steward provides targeted teaching to address the framework error. It is often helpful to offer an alternative path forward—as doing so opens the door to collaborative, shared decision-making and demonstrates shared investment in the patient’s outcome. Providing resources (eg, clinical guidelines or scientific articles) can also substantiate the steward’s recommendation and serve as affirmation and reassurance for providers, especially when patients are critically ill and clinical decisions may be guided by emotion and concern.

The open-ended and unbiased approach of the *advocacy-inquiry* statement sets the stage for a non-confrontational and targeted *identify-teach* dialogue, as illustrated in Table 1. By correctly identifying the framework error, the steward creates a concise and high-yield teaching opportunity—and avoids the pitfall of inadvertently teaching content that is already known. Most importantly, the *identify-teach* framework allows the steward to correct an underlying misunderstanding and behavioral habit—thus improving both the care of the individual patient and avoiding future errors related to the same issue.

**Table 1.** Hypothetical scenario incorporating the *advocacy-inquiry* and *listen-teach* approach in antimicrobial stewardship

Step	1: Advocacy	2: Inquiry	3: Identify	4: Teach
**Goal**	Highlight a perceived discrepancy by juxtaposing two conflicting facts using objective language	Probe this discrepancy with an open-ended question	Listen carefully to understand their rationale and identify the framework error that led to their decision	Provide targeted teaching to address the framework error and suggest an alternative path forward
**Clinical Example** A 55-yr-old patient with an indwelling Foley catheter is noted to have cloudy and malodorous urine. No fever, leukocytosis, or abdominal/flank pain is reported. A urinalysis and urine culture are ordered, and empiric ceftriaxone is planned.	*“I noticed this patient has a urine culture ordered, but the patient reports no urinary symptoms right now.”*	*“Can you share more about what triggered this urine culture?”*	Colleague A response: *“I think the patient’s cloudy and malodorous urine is an early sign of urinary tract infection.”* Framework error: Isolated foul-smelling and cloudy urine are not reliable signs of urinary tract infection^9^	*“Foul-smelling urine is an unreliable indicator of infection in catheterized patients, and it more likely reflects their hydration status and urea concentration in the urine. I’d suggest we monitor off antibiotics to see if any signs or symptoms of urinary tract infection develop.”*
			Colleague B response: *“I ordered it as a pre-operative culture to screen for asymptomatic bacteriuria; the patient is scheduled for pacemaker placement tomorrow.”* Framework error: Non-urologic surgeries do not require screening and treatment of asymptomatic bacteriuria^10^	*“Guidelines recommend pre-operative screening and treatment of asymptomatic bacteriuria only for urologic surgeries in which mucosal bleeding is expected. Even if this urine culture is positive, it would not require treatment; as a result, I don’t believe this testing would benefit the patient, and I think we could safely cancel it.”*

Though this framework is designed to create open dialogue and minimize defensiveness, clinical disagreement may remain even after targeted teaching. It is important to acknowledge that not every conversation will lead to success the first time, nor should that be a realistic expectation. The act of initiating a collegial conversation creates an opportunity for a thoughtful pause and may reiterate concepts that can carry momentum for the next conversation.

## Conclusion

Effective communication in clinical medicine requires initiating difficult conversations and providing unsolicited feedback to peers, both of which are crucial for ongoing professional growth and maintaining patient safety and high-quality care. To facilitate productive conversations, clinicians must create an open and psychologically safe atmosphere in which concise, targeted teaching can occur. The paired *advocacy-inquiry* statement followed by the *identify-teach* framework creates an organized approach to initiating such conversations and fostering productive relationships with colleagues across specialty and rank.
